# Clinical and Neurophysiological Evidence of Mononeuritis Multiplex During the COVID-19 Era

**DOI:** 10.7759/cureus.36853

**Published:** 2023-03-29

**Authors:** Nareen H Hasrat, Haithem J Kadhum, Ali R Hashim, Zaineb A Yakob, Hassan A Farid

**Affiliations:** 1 Neurophysiology, College of Medicine, University of Basrah, Basrah, IRQ; 2 Physiology, College of Medicine, University of Basrah, Basrah, IRQ; 3 Neurology, College of Medicine, University of Basrah, Basrah, IRQ; 4 Neurology, St. George's University of London, London, GBR

**Keywords:** neurophysiology, basrah, covid-19, multifocal mononeuropathy, mononeuritis multiplex

## Abstract

Infection with the novel coronavirus that causes coronavirus disease 2019 (COVID-19) results in a variety of clinical symptoms, including various neurological abnormalities. Peripheral nervous system symptoms, such as peripheral neuropathies, were often recorded in the medical literature, primarily as Guillain-Barré syndrome. Mononeuropathy multiplex is a multifocal axonal neuropathy commonly associated with vasculitis or connective tissue disease. Recent evidence about its associations with severe COVID-19 infection and intensive care unit hospitalization is being considered. A 58-year-old man with clinical and electrophysiological confirmation of mononeuropathy multiplex was reported during the peri-COVID-19 illness. He was treated with steroids and achieved a satisfactory response. Therefore, clinical and neurophysiological evaluation is recommended for any patient presenting with neurological manifestations following COVID-19 infection.

## Introduction

The emerging severe acute respiratory syndrome coronavirus-2 (SARS-CoV-2 virus) was first described in December 2019 as a cluster of severe pneumonia in Wuhan, China, then spread worldwide [1]. Although the main effect of this disease is on the respiratory system, some reports explain its affection for other systems, especially the nervous system. Mao et al. report that 36.4% of the patients infected with COVID-19 develop neurological manifestations, compromising the central nervous system in 24.8% of the cases as well as the peripheral nervous system in 8.9% of the cases, and 10.7% of the patients have musculoskeletal manifestations [2]. The local literature from Basrah, southern Iraq, showed that 60.7% of the patients involved in the study were documented to have neurological manifestations, and 10%-20% of them suffered from limb weakness and peripheral loss of sensation [3].

Mononeuritis multiplex is a multifocal neuropathy that does not display a root pattern or a length-dependent pattern. Numerous diseases, which are infectious, inflammatory, neoplastic, toxic, metabolic, and genetic, can emerge. However, a vasculitic origin accounts for most multifocal neuropathies' presenting patterns [4].

The damage to the vasa nervosum causes the abrupt or subacute onset of pain and a motor deficit, which is the hallmark of vasculitis-related neuropathy. Large nerve arterioles affected by vasculitis lead to sensory and motor disruption, neuropathic pain, and focal paralysis. After a few weeks, these findings may impact other nerves [5].

Axonal damage is diagnosed electrophysiologically by the evidence of an asymmetrical reduction in the amplitude of motor or sensory action potentials with average or slightly diminished conduction velocities in nerve conduction studies. Moreover, needle electromyography can detect membrane instability through positive, sharp waves and fibrillation [6].

This report aims to describe a rare complication in a patient with a confirmed diagnosis of COVID-19, whose clinical and electrophysiological characteristics suggest mononeuritis multiplex.

## Case presentation

A 58-year-old male from Basrah, southern Iraq, presented with a three-day history of shortness of breath and cough, preceded by a one-week history of sore throat and high-grade fever. He had no significant findings in his systemic review and had negative past medical and surgical histories. He was neither a smoker nor an alcoholic and was not on acute or chronic medications. On examination, he was conscious and oriented but looked tired, dyspneic, and tachypneic. His vital signs were as follows: blood pressure was equal to 125/80 mmHg, pulse rate was 120 beats per minute, core temperature was 39 degrees centigrade, and his respiratory rate was equal to 25 cycles per minute with an oxygen saturation of 70% on room air and of 91% with supplemental oxygen through a non-rebreather mask. His polymerase chain reaction (PCR) was positive for COVID-19 infection, and chest computed tomography (CT) also confirmed the presence of pneumonia with 70% lung involvement. The patient received the national therapeutic protocol for COVID-19, including the antiviral drug remdesivir, anti-coagulants such as subcutaneous low molecular weight heparin, steroids such as dexamethasone, and empirical antibiotics in the form of third-generation cephalosporin.

**Figure 1:**
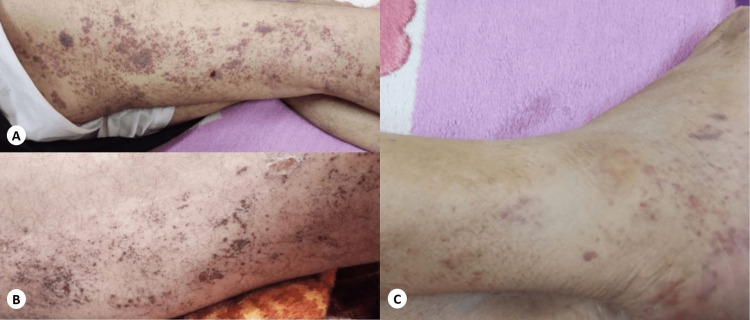
Vasculitic skin lesions observed on day 10 of admission (A) Posterolateral view of the thigh; (B) Magnified view of the posterior aspect of the thigh; (C) Lower leg and foot with vasculitic lesion eruption

Ten days after admission, the patient remained in the same condition despite treatment, with a deterioration of his oxygen saturation to 84% on oxygenation with a non-rebreather mask. The diagnosis of a secondary bacterial infection was confirmed with Klebsiella pneumonia, and the antibiotic choices were changed according to the culture and sensitivity results. During this time, a skin rash erupted suddenly. It was a strictly unilateral polymorphic skin lesion consisting of erythematous bluish macules, a small purpuric rash, and hemorrhagic vesicles scattered here and there, distributed at the lateral aspect of the right lower limb up to the little toe, as shown in Figure 1. The dermatologist's opinion was toward the diagnosis of a vasculitic skin lesion.

At the same time, a weakness was noticed in the right lower limb, and the power was equal to grade 3 with hypotonia and a decreased knee jerk with an absent ankle reflex. The patients also felt numbness and tingling sensations in both upper extremities. A pinprick sensation was lost in the left upper arm, but the sensory examination of the other limbs was normal. The brain image revealed no signs of acute ischemia or hemorrhage, and the doppler lower limb ultrasound scan revealed no signs of thrombosis. A new set of blood tests was ordered, which showed a marked deterioration of inflammatory biomarkers (erythrocyte semination rate, lactate dehydrogenase, serum ferritin, and C-reactive protein). The diagnosis of a cytokine storm was considered. The laboratory studies are described in Table 1.

**Table 1:** The patient's laboratory investigations on admission, after 10 days of admission, and on discharge g/dl: gram per deciliter; m3: cubic milliliter; %: percentage; mg/L: milligram per liter; mm/hr: millimeter per hour; ng/ml: nanogram per milliliter; IU/L: international unit per liter; mg/dl: milligram per deciliter; U/L: unit per liter

Biomarker	On admission	On day 10	On discharge	Reference range *
Hemoglobin level (g/dl)	14.9	14.3	13.8	12.0 – 16.0
White blood cells (cell/m^3^)	11.000	19.000	9.000	4.000 - 11.000
Neutrophile (%)	85.6	79.8	71.8	40 – 60
Lymphocyte (%)	12.6	19.4	25.3	20 – 40
Platelets (cell/m^3^)	118	188	160	150 - 400
C-reactive protein (mg/L)	24	74	10	0 - 10
Erythrocyte semination rate (mm/hr)	30	62	21	0 – 20
Serum ferritin (ng/ml)	496	860	389	24 – 285
Lactate dehydrogenase (IU/L)	397	932	278	140 - 280
D-dimer (ng/ml)	2	10	4	0 – 0.5
Blood urea (mg/dl)	60	56	40	7 – 30
Serum creatinine (mg/dl)	0.67	0.79	0.63	0.7 – 1.2
Random blood sugar (mg/dl)	133	210	270	> 200
Alanine aminotransferase (U/L)	36	51	43	< 42
Aspartate aminotransferase (U/L)	14	35	31	< 37
* The reference ranges are of the laboratory devices				

The patient was treated with the immunomodulatory drug known as tocilizumab and kept on continuous positive airway pressure (CPAP) for seven days with the supportive treatment lines that have already been mentioned. A gradual improvement was noticed with a decline in the inflammatory markers and improvements in the oxygen saturation and respiratory status during the third week of admission. The patient was discharged after three weeks of admission on oxygen due to the development of lung fibrosis that was diagnosed with a new chest CT scan, in addition to persistent weakness and painful limbs with numbness and tingling sensations.

A thorough neurological workup was done for the patient, including a vasculitis panel and a connective tissue screen, which were negative for any antibodies, including antinuclear antibodies (ANA), anti-double-stranded DNA (dsDNA), anti-neutrophil cytoplasmic antibody (ANCA), anti-cardiolipin antibodies, lupus anticoagulant, and the extractable nuclear antigen (ENA) panel (anti-Ro, anti-La, anti-Sm, anti-RNP, anti-Jo-1, anti-Scl70, anti-centromere). Also, a whole-spine magnetic resonance imaging (MRI) was performed, which revealed chronic degenerative changes affecting all the disc spaces but with no significant compression on nerve roots or canal stenosis.

A detailed electrodiagnostic study (EDX) confirmed the diagnosis of mononeuritis multiplex. The nerve conduction study is described in Tables 2-4.

**Table 2:** The motor nerve conduction study of the patient * The recording muscles are abductor pollicis brevis for the median nerve, abductor digiti minimi for the ulnar nerve, extensor digitorum brevis for peroneal nerve, and abductor hallucis brevis for tibial nerve. ms: millisecond; mV: millivolts; m/s: meter per second

Motor nerve conduction study									
Nerve *	Distal latency (ms)		Reference range	Amplitude (mV)		Reference range	Conduction velocity (m/s)		Reference range
	Right	Left		Right	Left		Right	Left	
Median	3.6	3.4	≤ 4.4	6.6	7.1	≥ 4	51.8	54.9	≥ 49
Ulnar	3.6	3.1	≤ 3.3	5.8	7.0	≥ 6	68.4	62.0	≥ 49
Peroneal	4.4	4.6	≤ 6.6	1.3	2.5	≥ 2	44.9	41.1	≥ 44
Tibial	4.5	4.6	≤ 5.8	0.9	1.9	≥ 4	50.9	51.7	≥ 41

**Table 3:** The study of the late responses ms: millisecond

Late responses			
Site	Latency (ms)		Reference range
	Right	Left	
Ulnar F wave	30.7	29.5	≤ 32
Tibial F wave	48.9	49.6	≤ 56
H reflex (ankle )	29.8	30.1	≤ 32

**Table 4:** The sensory nerve conduction study of the patient ms: millisecond; μV: microvolts; m/s: meter per second

Sensory nerve conduction study									
Nerve	Latency (ms)		Reference range	Amplitude (μV)		Reference range	Conduction velocity (m/s)		Reference range
	Right	Left		Right	Left		Right	Left	
Ulnar	3.2	3.2	≤ 3.1	16.9	18.8	≥ 17	43.5	43.5	≥ 50
Sural	3.3	3.1	≤ 4.4	5.5	13.0	≥ 6.0	44.6	42.9	≥ 40

The motor nerve conduction study of the right peroneal nerve showed low amplitude recorded from the extensor digitorum brevis and tibialis anterior as compared to the left side, in addition to the very low amplitude of the right and left tibial nerve. The right sural nerve's sensory response showed low amplitude compared to the left side. The motor nerve conduction studies of the right and left ulnar nerves showed low amplitude of the right ulnar nerve compared to the left side with normal latency and conduction velocity. Also, there was a normal distal motor latency of the right and left median nerves with normal amplitude and conduction velocity. There was a prolonged sensory response of the right median nerve.

Needle electromyography (EMG) of the right tibialis anterior, left extensor halluces longus, right and left vastus medialis, and right and left first dorsal interossei revealed no spontaneous activity but a large-amplitude, long-duration motor unit action potential (MUAP) with a reduced recruitment pattern in the extensor halluces longus and tibialis anterior (Table 5).

**Table 5:** The needle electromyography of the patient MUAP: motor unit action potential

Needle electromyography				
Finding	First dorsal interosseous	Tibialis anterior	Vastus medialis	Extensor hallucis
Spontaneous activity	Nil	Nil	Nil	Nil
MUAP duration	Normal	Long	Normal	Long
MUAP amplitude	Normal	Large	Normal	Large
Recruitment	Normal	Reduced	Normal	Reduced
Number of phases	Normal	Polyphasic	Normal	Polyphasic

The EDX study showed evidence of chronic bilateral sensory-motor neuropathy of moderate degree affecting the lower and upper limbs with asymmetrical distribution (sensory and motor fibers on the right side were affected more than those on the left side). The main pathology is axonal degeneration with signs of chronic re-innervation. The possibility of mononeuritis multiplex is highly suggestive.

Unfortunately, the patient refused to have a nerve biopsy. However, a therapeutic trial of prednisolone was started. After three months of treatment, the patient's condition improved, and the weakness declined, with a marked decrease in pain and numbness.

## Discussion

With the COVID-19 pandemic spread, most of the literature highlights the acute post-infectious polyneuropathy, formerly known as Guillain-Barré syndrome (GBS), as the literature reported a 5.41-fold increase in GBS cases in 2020 compared to 2017-2019 [7]. According to a recent cohort study from Basrah, the prevalence of GBS and chronic inflammatory demyelinating polyneuropathy (CIDP) is six times higher in the COVID-19 population compared to the non-COVID-19 sample [8]. However, mononeuritis multiplex was found in a large proportion of the patients (11 of 69, 16%) with severe COVID-19 admitted to the intensive care units [9].

One of the frequently described traits of various types of virulent coronaviruses, including the severe acute respiratory syndrome coronavirus (SARS-CoV) in 2002 and the Middle East respiratory syndrome coronavirus (MERS-CoV) in 2012, is neurotropism [10]. SARS-CoV-2 may target endothelium, glial cells, and neurons because these tissues have been found to have angiotensin-converting enzyme 2 (ACE2) receptors [5].

Post-mortem studies have confirmed that vasculitis is prominent in many tissues in patients dying from COVID-19 [11]. The etiology may be related to an endotheliopathy [12] resulting from the cytokine storm or perhaps microthrombi [13]. Still, the possibility of immune-mediated vasculitis neuropathy is another suggested mechanism [14], as in other related COVID-19 neuropathies. In our patient, all the above-mentioned possible mechanisms might have played a role, as he had a diagnosis of critical COVID-19 with cytokine storm documented by the clinical manifestation and the elevation of inflammatory markers, as well as the possibility of hypercoagulability despite the fact that during COVID-19, D-dimer might rise due to the hyperinflammatory storm as an acute phase reactant and not necessarily due to thrombotic or embolic phenomena [15].

In severe situations, the SARS-CoV-2 virus causes abnormal immunological activation and inflammation similar to macrophage activation syndrome. The reported case in this article was already diagnosed with a cytokine storm and an elevated interleukin-6 level. It is well known that interleukin-6 levels in COVID-19 patients have significantly increased, according to preliminary investigations [16]. It has been hypothesized that this cytokine storm contributes to the development of some vasculitides, such as Kawasaki disease, because of how it affects endothelial cells. It increases the number of adhesion molecules on the cell surfaces, enhancing lymphocyte adhesion to the cells, and encouraging endothelial damage [17].

According to Carberry and colleagues, a preceding COVID-19 infection is linked to the emergence of a systemic neuropathy that shows up as an asymmetric sensorimotor loss. They outline four mononeuropathy multiplex patients identified following the COVID-19 infection [18].

On the other hand, a low platelet count was observed in this patient. There are relatively few publications in the scientific literature highlighting the link between thrombocytopenia and mononeuritis multiplex. It has been shown that acquired amegakaryocytic thrombocytopenia is related to neuropathy. Several pathogenetic processes have been proposed, but the most widely accepted pathological explanation is an immune-mediated mechanism owing to an autoantibody that prevents the effect of endogenous thrombopoietin on megakaryocytopoiesis. The neuropathy in our case was probably triggered by a thrombocytopenia-related nerve hemorrhage. In idiopathic thrombocytopenia purpura, an intraneural hematoma under the epineurium and between the fascicles was shown to be the source of a similar neuropathy. However, there is a paucity of reports that discuss the association between acquired amegakaryocytic thrombocytopenia and mononeuritis multiplex [19].

It is well known that the severe mononeuritis multiplex justified additional treatment with high doses of glucocorticoids, cyclophosphamide, and then rituximab [20]. Regarding our patient, he was already on intravenous steroids and tocilizumab during hospitalization and was kept on oral steroids even after discharge, and there was a noticeable improvement. This also strongly confirms the diagnosis of vasculitis-related neuropathy.

## Conclusions

COVID-19-related complications are diverse, can be misleading, and are difficult to diagnose. A thorough evaluation of the systemic features and neurological manifestations, especially for any patient with severe COVID-19, is highly recommended. Even though mononeuropathy multiplex is a rare occurrence, with only a few cases reported worldwide, it is mandatory to highlight such a complication as it has a poor outcome and can lead to permanent disability. Further studies are required to look for the potential neurological complications of COVID-19 in both the short-term and long-term eras.
